# Excitonic Mechanisms of Stimulated Emission in Low-Threshold ZnO Microrod Lasers with Whispering Gallery Modes

**DOI:** 10.3390/ma15248723

**Published:** 2022-12-07

**Authors:** Andrey P. Tarasov, Arsen E. Muslimov, Vladimir M. Kanevsky

**Affiliations:** Shubnikov Institute of Crystallography, Federal Scientific Research Centre “Crystallography and Photonics” of Russian Academy of Sciences, 119333 Moscow, Russia

**Keywords:** ZnO, microrod, excitonic lasing, low-threshold lasing, optical gain, stimulated emission, whispering gallery modes, exciton-electron scattering, exciton-exciton scattering, two-phonon assisted exciton recombination

## Abstract

Whispering gallery mode (WGM) ZnO microlasers gain attention due to their high *Q*-factors and ability to provide low-threshold near-UV lasing. However, a detailed understanding of the optical gain mechanisms in such structures has not yet been achieved. In this work, we study the mechanisms of stimulated emission (SE) in hexagonal ZnO microrods, demonstrating high-performance WGM lasing with thresholds down to 10–20 kW/cm^2^ and *Q*-factors up to ~3500. The observed SE with a maximum in the range of 3.11–3.17 eV at room temperature exhibits a characteristic redshift upon increasing photoexcitation intensity, which is often attributed to direct recombination in the inverted electron-hole plasma (EHP). We show that the main contribution to room-temperature SE in the microrods studied, at least for near-threshold excitation intensities, is made by inelastic exciton-electron scattering rather than EHP. The shape and perfection of crystals play an important role in the excitation of this emission. At lower temperatures, two competing gain mechanisms take place: exciton-electron scattering and two-phonon assisted exciton recombination. The latter forms emission with a maximum in the region near ~3.17 eV at room temperature without a significant spectral shift, which was observed only from weakly faceted ZnO microcrystals in this study.

## 1. Introduction

Efficient optical nano/microresonators and miniature laser sources are in demand today in many areas, including biological and physical sensors, optical microcircuits (including all-optical ones), medical diagnostics, and target therapy [1,2,3,4,5,6,7]. Zinc oxide (ZnO) structures are a good candidate for the development of nano and microlasers operating in the near UV range. Possessing unique optical properties, including a wide and direct bandgap, high exciton binding energy, high optical gain and refractive index, ZnO is also characterized by a relatively low fabrication cost and the ease of obtaining a huge variety of nano- and microstructures [8,9,10,11].

Speaking about the efficiency of ZnO laser structures in terms of achieving low lasing thresholds and high optical *Q*-factors, the most promising ones are those supporting whispering gallery modes (WGMs). Due to total internal reflection and, consequently, low optical losses, these microlasers demonstrate much better energy efficiency compared to their Fabry-Perot counterparts [12,13]. The study of the fundamental properties of WGM microlasers, as well as their practical applications, is an important task of modern semiconductor physics and materials science in terms of obtaining functional objects on the micro and nanoscale. However, the mechanisms of stimulated emission (SE) in ZnO WGM microlasers, especially at room temperature (RT), remain insufficiently clear today, and contradictions often occur among existing works.

In general, despite the existence of different opinions and interpretations of the nature of optical gain in ZnO structures with WGMs, with some exceptions, they can be divided into two blocks depending on the spectral behavior of the laser band. Under optical UV excitation of ZnO microcrystals of different sizes, the maximum of the WGM lasing profile usually appears in the region of 3.14–3.20 eV (387–395 nm) at RT, depending on the properties of a specific structure and excitation conditions. As the pumping increases, the behavior of the lasing band can be different. In one case, the maximum of the gain profile can be intensively shifted to the long wavelength, leading to a redistribution of the intensities of the laser peaks and, as a result, a redshift of the entire lasing band. This situation is described, e.g., in [12,14,15,16,17]. In another case, such a shift may not be so pronounced or may not appear at all [12,14,15,18,19,20,21]. In the case of a strong redshift, it is generally believed that the optical gain is due to direct recombination in the inverted electron-hole plasma (EHP) [12,14,15,16], except in some cases where the excitonic nature is stated but without further details (for example, [14]). The redshift in this case is attributed to bandgap renormalization. In the second case, when the shift is absent, excitonic mechanisms are usually considered [14,15,18,19,20,21]. In particular, the process of inelastic exciton-exciton scattering [19,20] or two-phonon assisted emission of excitons [21] is claimed to be responsible for SE. At the same time, the lack of a clear understanding of the optical gain mechanisms in ZnO WGM microlasers leads to limitations in their practical application, including control, modification, and optimization of the laser parameters.

In this work, we study the nature of optical gain in the near UV range in WGM ZnO microlasers. Large-diameter ZnO microrods demonstrating low-threshold WGM lasing with a typical shifting profile, preliminarily studied in [22], were chosen as the object of the study. It was shown in [22] that the highest *Q*-factor (more than 3000) and the lowest thresholds (down to 10–20 kW/cm^2^) were demonstrated by microrods with diameters of more than 10 μm, characterized by directional growth. As a result of the present study, the SE mechanisms at RT and lower temperatures in such microlasers were elucidated. We showed that the main contributor to the RT optical gain with a redshifting profile in the microrods studied is the exciton-electron scattering process rather than direct recombination in the EHP. It was found that the perfection of the microcrystal plays an important role in the formation of such emission. At lower temperatures, the simultaneous participation of various processes leading to optical gain is possible. In particular, the contribution of two-phonon assisted free exciton recombination to the low-temperature SE of microcrystals was described, as was the manifestation of this emission at RT.

## 2. Materials and Methods

**Figure 1:**
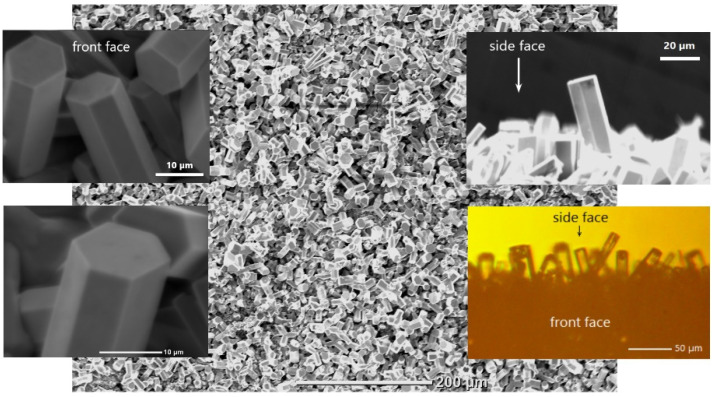
Microphotographs of an ensemble of ZnO microcrystals. The left insets show SEM images of ZnO microcrystals formed on the front face of the sample. The right insets show images obtained by SEM (upper) and optical microscopy (bottom) of the edge region of the sample—individual microrods oriented in space are seen on the side face of the sample.

ZnO microcrystals were fabricated by pyrolytic carbothermal synthesis [22]. Figure 1 shows scanning electron microscopy (SEM) (JSM-6000PLUS, JEOL, Japan) and optical microscopy (MBI-15, LOMO, Russia/USSR) images of the sample. Microcrystals represent hexagonal prisms with clearly defined side faces and flat, smooth ends perpendicular to the main *c* axis. For this study, we used individual microrods formed near the edge of the sample and, particularly, at the side faces of the substrate (see right insets in Figure 1). As was shown in [22], these microrods demonstrate low-threshold WGM lasing with relatively high *Q*-factors. The density of such lasing microrods on the substrate was quite low, and their directional growth predominated. Their lengths and diameters were up to 50 and 20 μm, respectively. All this made it possible to observe lasing from individual microrods.

Excitation of ZnO radiation was performed with a 3rd harmonic (355 nm) of a *Q*-switched Nd:YAG laser (Lotis TII, Belarus). The pulse duration and pulse repetition frequency were 10 ns and 15 Hz, respectively. The size of an excitation spot on the samples was ~100 μm. The sample’s radiation was registered by the use of a Peltier-cooled charge-coupled device camera (Videoscan, Russia) placed behind the exit slit of a monochromator. To elucidate the SE mechanisms in the fabricated crystals, we studied their near-band-edge (NBE) emission as a function of temperature at different photoexcitation intensities. Two emission regimes were realized, when lasing and amplified spontaneous emission (ASE) were observed from ZnO crystals, respectively.

More details about growth mechanisms, experimental research methods, and the equipment used can be found elsewhere [22,23,24].

## 3. Results

**Figure 2:**
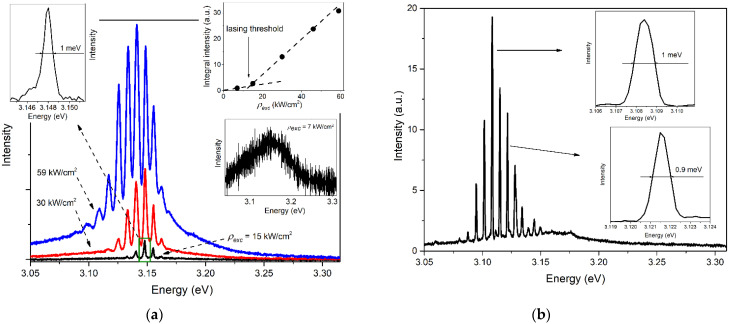
RT lasing spectra of the individual ZnO microrods: (**a**) the evolution of the lasing spectra at different $\rho_{exc}$; the left inset shows the laser peak at 3.148 eV; the right bottom inset shows the spectrum at $\rho_{exc}$ = 7 kW/cm^2^; the right upper inset shows the dependence of the intensity in the maximum of the laser peak at 3.148 eV on $\rho_{exc}$; (**b**) the lasing spectrum recorded at $\rho_{exc}$ = 520 kW/cm^2^.

For study in the lasing regime, a region of the sample demonstrating low-threshold WGM lasing from a single large-diameter microrod was selected. A typical picture of the evolution of the lasing spectra of such a microrod at different excitation power densities $\rho_{exc}$ at RT is shown in Figure 2a. At the initial excitation intensity, the NBE emission spectrum contains a fairly wide band, with the maximum at ~3.16 eV and the full width at half maximum (FWHM) of ~140 meV (the right bottom inset in Figure 2a). As an excitation intensity increases, narrow peaks appear near the maximum of the wide emission band, indicating the onset of lasing. The right upper inset in Figure 2a plots the dependence of the intensity at the maximum of one of the laser peaks (at 3.148 eV) on $\rho_{exc}$. The characteristic kink in this dependence at $\rho_{exc}$~13 kW/cm^2^ corresponds to the lasing threshold for this optical mode.

As was shown in [22], where optical modes in these microrods were studied, lasing can be described by the plane wave model for the WGM cavity in this case. In particular, WGM modes with TE polarization are excited, the spectral positions of which are given by the expression:

(1) $$E_{TE-WGM}=\frac{2hc}{3\sqrt{3}Dn\left(E\right)}\left[N+\frac{6}{\pi }tan^{-1}\left(n\left(E\right)\sqrt{3n{\left(E\right)}^{2}-4}\right)\right],$$

where *N* is the mode number, *E* is the photon energy, $n\left(E\right)$ is a reflection index, *D* is the hexagonal cavity’s diameter, and *h* is the Planck constant [14,25]. In the case under study, the use of the refractive index dispersion relation, which was obtained in [13] for ZnO microcrystals fabricated by the same method, allows us to estimate the diameter of the lasing microrod as *D* ≈ 14 µm [22]. The left inset of Figure 2a shows a spectral region with the most intense laser line, with a maximum at *E_m_* = 3.148 eV at $\rho_{exc}$ = 15 kW/cm^2^. The line’s full width at half maximum, *γ*_1/2_ ≈ 1 meV, allows us to estimate the corresponding *Q*-factor as *Q* = *E_m_*/*γ*_1/2_, which gives *Q* ≈ 3150.

It is important to note that, despite the possibility of obtaining low laser thresholds, such WGM lasing was observed in the present study at much higher excitation intensities. For example, Figure 2b shows the lasing spectrum of one of the studied ZnO microrods at $\rho_{exc}$ = 520 kW/cm^2^. It can be seen that the entire lasing band is shifted towards longer wavelengths as compared to the spectra in Figure 2a, recorded at much lower excitation intensities. The most intense laser peak in the spectrum in Figure 2b is at 3.1085 eV with a FWHM of 1 meV. Some neighboring peaks have a lower FWHM of ~0.9 eV. For example, the *Q*-factor in the case of a peak at 3.1215 eV is *Q* ≈ 3470.

One of the features of lasing observed was a redshift of the lasing band with increasing photoexcitation intensity. This is clearly seen in Figure 2: an increase in pumping leads to the appearance of new laser peaks, mostly on the low-energy side, and a redistribution of intensities between the peaks with a relative enhancement of the lower-energy ones. Thus, we observe a typical case of WGM lasing in ZnO when the increase in pumping is accompanied by a redshift of the optical gain profile (see Section 1).

**Figure 3:**
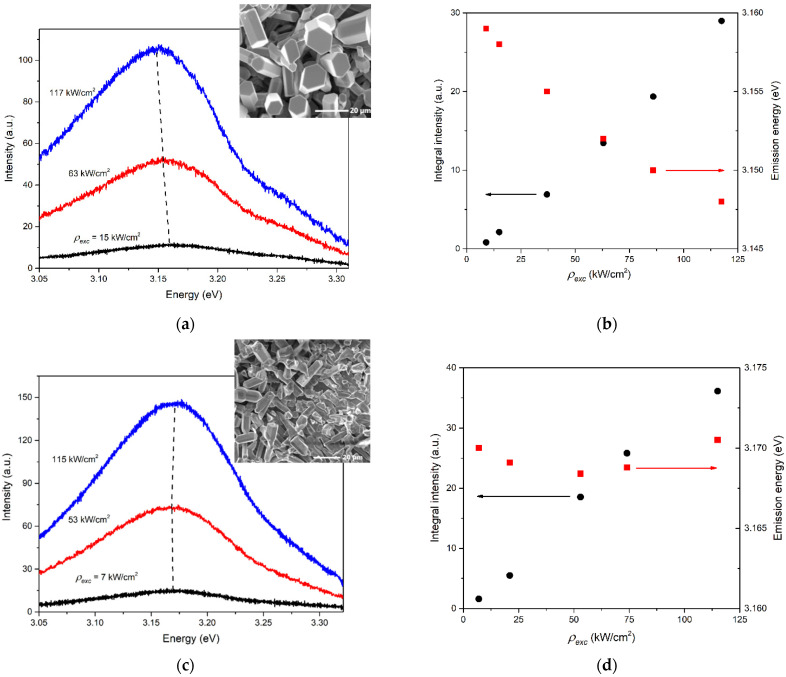
RT ASE of the sample: (**a**,**c**) type I (**a**) and type II (**c**) emission spectra at different $\rho_{exc}$; (**b**,**d**) integral intensity (black circles) and emission energy at maximum intensity (red squares) vs. $\rho_{exc}$ for the type I (**b**) and type II (**d**) emission spectra. The insets in (**a**,**c**) show the sample’s regions with well (**a**) and weakly (**c**) faceted microcrystals.

For study in the ASE regime, the sample’s regions that show two different SE types at RT were selected. In the first case, a region with well-faceted ZnO microcrystals, whose lasing thresholds were higher than in the case of individual microrods, was examined (the inset in Figure 3a shows an example of such a region). The ASE band of this region exhibited a superlinear growth and behavior in general similar to the lasing band at RT; in particular, it intensively shifted with the increase of $\rho_{exc}$ (Figure 3a,b). For such a band and the corresponding spectrum, the “type I” designation was used. In the second case, the ASE band observed at RT did not shift significantly with increasing $\rho_{exc}$, being characterized by its maximum position in the region near ~3.17 eV (Figure 3c,d). At the same time, it first exhibited a superlinear growth and then saturation (Figure 3d). “Type II” designation was used to identify this band and its corresponding emission spectra. To observe type II emission, we used the sample’s regions, where weakly faceted microcrystals were formed and well-faceted laser crystals were almost completely absent (see the inset in Figure 3c).

**Figure 4:**
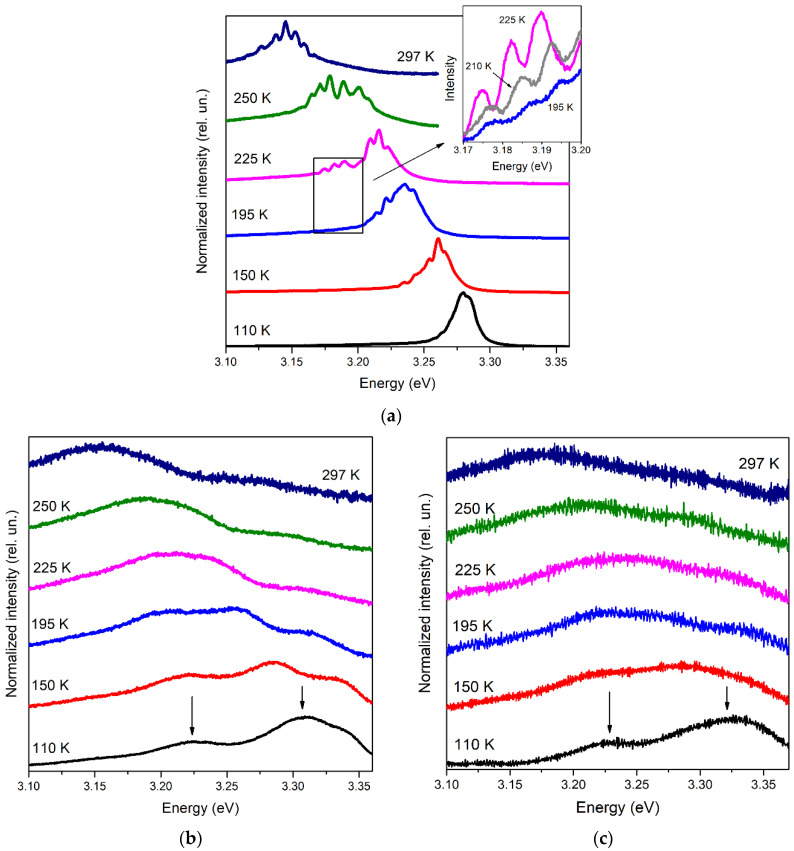
NBE emission spectra of the sample at different temperatures in the case of: (**a**) lasing at $\rho_{exc}$ = 45 kW/cm^2^; (**b**) type I emission at $\rho_{exc}$ = 15 kW/cm^2^; (**c**) type II emission at $\rho_{exc}$ = 6 kW/cm^2^. Spectra are normalized at maximum intensity and displaced vertically for clarity. The arrows in (**b**,**c**) indicate the traced bands (see the text below and Figure 5).

Figure 4 illustrates the behavior of the NBE emission spectra in the lasing (Figure 4a) and ASE (Figure 4b,c) regimes at different temperatures.

Studying NBE emission at different excitation intensities turned out to be essential since it allowed us to identify emission types with different thresholds and understand whether they could be stimulated. In this regard, the behavior of the lasing spectra with increasing temperature was extremely indicative (Figure 4a). At a lower temperature, when the gain profile is narrow, the position and width of the SE band strongly depend on the mode structure of the laser crystal and are determined by the optical mode closest to the gain maximum. As the temperature increases and the gain profile broadens, individual laser peaks become visible within this specific SE band.

Beginning at ~190 K, mode enhancement on the low-energy side of this SE band noticeably increases. The laser peaks appearing in this region become more and more intense with rising temperature (see the inset in Figure 4a). It becomes clear that a second, lower-energy SE band appears and begins to compete with the first one. Both bands, represented by two sets of laser peaks, are clearly distinguishable up to *T*~250 K, after which one broad emission (lasing) band is formed. Exhibiting a general broadening and redshift, the lasing spectrum retains this appearance up to RT.

In addition to the redshift of the gain profile, a redshift of laser peaks was observed with increasing temperature (see the inset in Figure 4a, for example), which was about 0.1–0.2 meV/K depending on the spectral range and temperature. Approximately the same order shift of the laser peaks was also obtained for ZnO microcrystals with an increase in temperature above RT [26]. This redshift is mainly a consequence of the increase in the refractive index with rising temperature [27].

Giving the possibility to reveal individual SE bands, on the one hand, the laser effect strongly modifies the NBE emission spectrum and shifts and distorts the emission bands, making it difficult to analyze their nature, on the other hand. In this case, sub-threshold studies are useful. The evolution of the NBE emission spectra of types I and II with temperature is shown in Figure 4b,c, respectively. Here, two bands, which converge with increasing temperature, are traced from low temperatures to ~200 K (arrows in Figure 4b,c). With a further increase in temperature, the bands merge, and then only one spectral maximum can be observed.

**Figure 5:**
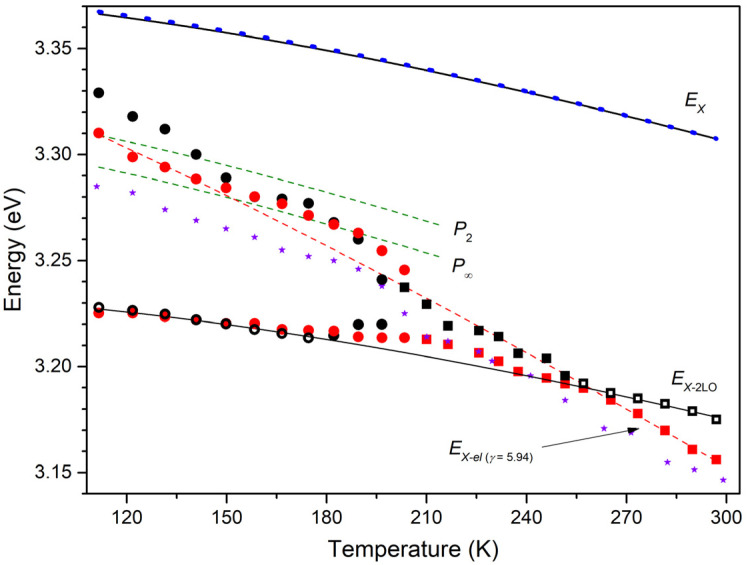
Temperature dependence of spectral bands’ maxima. Red and black symbols correspond to the experimentally observed emission bands in type I and type II spectra, respectively; circles and squares denote separate and merged emission bands (open symbols were used for fitting); stars correspond to the lasing band. Solid lines are fitted dependences for *E_X_*(*T*) (upper) and *E_X-_*_2LO_(*T*) (bottom). Dashed lines indicate *P*_2_(*T*) and *P*_∞_(*T*) (4) (green), and *E_X-el_* (*T*) (5) with *γ* = 5.94 (red). *E_X_*(*T*) from [28] indicated with a blue dotted line.

The obtained temperature dependences of the emission bands’ maxima are collected in Figure 5 (symbols). One can see that the behavior of the traced bands is similar between the spectra of type I and type II below *T*~250–260 K. At higher temperatures, up to RT, the spectral behavior of the single emission maximum differs between the spectra of the two types, which results in the difference in the optical gain type at RT.

The temperature behavior of the lasing band maximum is also shown in Figure 5 (stars). In this case, up to *T*~190 K, i.e., before the appearance of two sets of laser peaks, the maximum of the single lasing band was used to trace the band’s energy. Above ~190 K, due to the difficulty of assigning a particular laser peak to one or another type of SE, the band’s position was estimated as an intensity-weighted average over the positions of all peaks [29]:$\;E_{max}=\sum_{n}E_{n}\cdot I_{n}/\sum_{n}I_{n}$, where $E_{n}$ and $I_{n}$ are the energy and the intensity of the *n*-th laser peak, correspondingly. Despite the lower-energy position of the lasing band as compared to the maximum of the type I spectrum, which is ascribed mainly to the increase in the exciton effective temperature, there is an obvious correlation between them. Moreover, as the photoexcitation intensity approaches the lasing threshold, both temperature dependences tend to merge, suggesting the same gain nature.

To interpret the observed bands, it is necessary to determine the bandgap energy $E_{g}\left(T\right)$ and/or exciton recombination energy $E_{X}\left(T\right)$ in the crystals under study. The true $E_{g}\left(T\right)$ (rather than effective or optical ones) is often difficult to determine correctly, particularly from the transmission or reflection spectra, since they may be strongly affected by both shallow and deep defect energy levels [30]. In this sense, the use of $E_{X}\left(T\right)$ obtained experimentally is a good way to indirectly determine $E_{g}\left(T\right)$ for specific samples, assuming the exciton binding energy $E_{b}\;$(60 meV in ZnO) to be constant [28]. In the cases where free exciton emission is difficult to distinguish, one can try to track the temperature dependences of other emission channels; for example, *X*-LO is used in [31].

**Figure 6:**
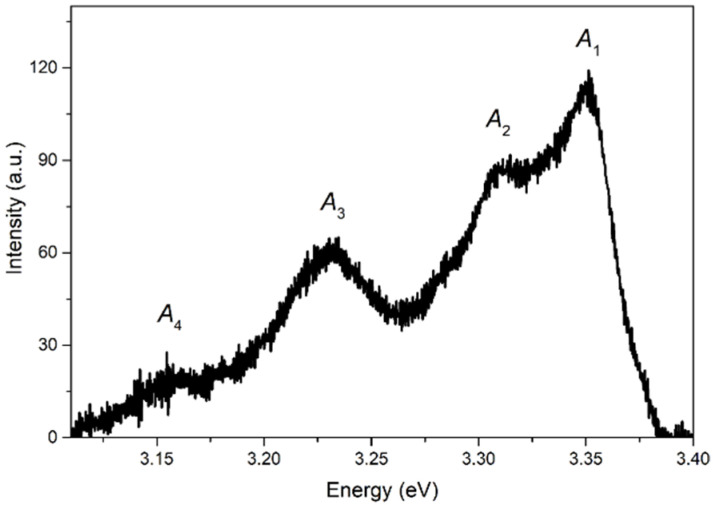
NBE emission spectrum of the sample at *T*~80 K.

Figure 6 shows the spontaneous emission spectrum recorded at *T*~80 K in the sample’s region containing weakly faceted crystals. To minimize SE effects, the lowest excitation intensity in this study ($\rho_{exc}$~2 kW/cm^2^) was used, along with large signal accumulation. The spectrum clearly shows several bands with maxima at 3.350 eV, 3.309 eV, 3.233 eV, and 3.161 eV, designated as *A*_1_, *A*_2_, *A*_3_, and *A*_4_, respectively. The *A*_1_ band is due to the emission of defect-bound excitons (*BX*) [28,32,33]. The *A*_2_ band is associated with a mixture of the first phonon replica of the free exciton recombination radiation (*X*-LO) [32,33,34] and emission associated with the surface defect states [34,35,36,37,38], such as free-to-bound transitions [34,35] and surface exciton recombination [36,37]; the contribution of donor-acceptor pairs [39,40] cannot be excluded either. Assuming that in such quite large microcrystals surface states do not prevail in emission as compared to, e.g., nanostructures possessing a high surface-to-volume ratio [38], we attribute the *A*_3_ and *A*_4_ bands spaced from *A*_2_ by 77 and 148 meV, respectively, to the second (*X*-2LO) and third (*X*-3LO) phonon replicas of free exciton emission (LO-phonon energy $E_{\mathrm{LO}}$ is 72 meV in ZnO) [32,33,34].

Thus, in our case, free exciton emission is not distinguishable in low temperature spectra. In addition, there is a strong overlap of *X*-LO and *BX* bands and the possible presence of other radiative channels in the spectral region of *X*-LO, which among others causes a slight increase in spacing between *A*_3,4_ and *A*_2_ bands. Taking this into account and also the fact that the *X*-LO band often merges with free exciton emission at RT [28,41], we decided to rely on the data for the *X*-2LO band. The latter is traced in the ASE spectra of both types at low temperatures (“low-energy” arrow in Figure 4), and, as the analysis showed, up to RT in the type II spectra (see Figure 5), which turned out to be essential in this study. Using the spectral position of the *X*-2LO band $E_{X-2\mathrm{LO}}\left(T\right)$, one can obtain $E_{X}\left(T\right)$ from the relation [32,42]:

(2) $$E_{X}\left(T\right)=E_{X-2\mathrm{LO}}\left(T\right)+2E_{\mathrm{LO}}-\frac{1}{2}k_{B}T,$$

where $k_{B}$ is the Boltzmann constant.

The obtained dependence (2) was fitted using Varshni’s formula [43]:

(3) $$E_{X}\left(T\right)=E_{g}\left(0\right)-E_{b}-\alpha T^{2}{\left(T+\beta \right)}^{-1},$$

where *α* and *β* are constants, $E_{g}\left(0\right)$ is the bandgap energy at *T* = 0. The obtained fitting parameters are $E_{g}\left(0\right)-E_{b}$ = 3.378 eV, *α* = 8.3 × 10^−4^ eV·K^−1^, *β* = 734 K. The resulting curve for $E_{X}\left(T\right)$ is plotted in Figure 5. By a dotted curve, we also plotted $E_{X}\left(T\right)$ obtained in [28], Equation (1), for bulk ZnO crystals—these data are often used in the analysis of the ZnO properties. It can be seen that the results are quite close, which is probably a consequence of the dimension factor: sufficiently large microcrystals exhibit the bulk ZnO properties. In particular, we obtain $E_{g}$ ≅ 3.37 eV at RT for the microcrystals studied in this research, as in [28].

$E_{X}\left(T\right)$ obtained from (3) allows modeling the temperature behavior of emissions of several types that can be stimulated in ZnO for the particular sample. The analysis showed that, in addition to *X*-2LO, the processes that most closely describe SE observed in the experiment are exciton-exciton (*X*-*X*) and exciton-electron (*X*-*el*) scattering, which cause radiative recombination of a free exciton after inelastic scattering by another exciton and a free electron, respectively [42,44,45,46].

The spectral position of the so-called *P*-band resulting from the *X-X* scattering is described with the equation:

(4) $$P_{n}\left(T\right)=E_{X}\left(T\right)-E_{b}\left(1-\frac{1}{n^{2}}\right)-\frac{3}{2}k_{B}T,$$

where *n* stands for the excited state of one of the scattered excitons [41,47].

A photon emitted upon *X-el* scattering has an energy of

(5) $$E_{X-el}\left(T\right)=E_{X}\left(T\right)-\gamma k_{B}T,$$

where *γ* is the coefficient depending on the ratio of the effective masses of the exciton and electron [42,44,45,46]. A characteristic feature of this type of emission is the intersection of the zero of the difference $E_{X}\left(T\right)-E_{X-el}\left(T\right)$ at *T* = 0.

**Figure 7:**
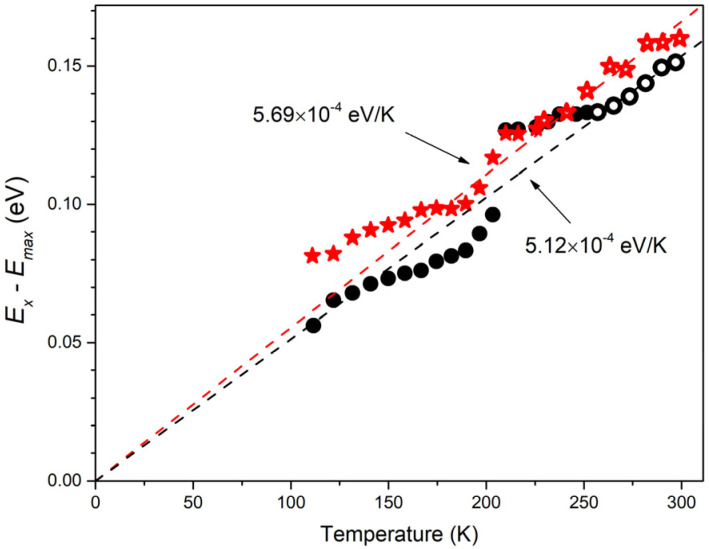
The energy differences between *E_X_*(*T*) and maxima of type I spectrum (black circles) and lasing spectrum (red stars) as a function of temperature; corresponding linear fitting (open symbols used) are shown by the dashed line extended to *T* = 0.

Figure 7 shows the temperature behavior of the difference of $E_{X}\left(T\right)$ obtained from (3) and the maximum energy $E_{max}\left(T\right)$ in the type I spectrum, determined up to *T*~200 K by the high-energy component. It can be seen that the dependence is linear in the range from *T*~250 K to RT, and the approximation of the line is well extrapolated to zero of coordinate. This line is described by the equation: $E_{X}\left(T\right)-E_{max}\left(T\right)=a\cdot T$ with *a* = 5.12 × 10^−4^ eV/K. This corresponds to the *X-el* process (5) with *γ* = 5.94. This value is consistent with the literature data [42,48,49,50], but slightly less than some of them. For example, in [49,50], where random lasing in ZnO disordered media was studied as a result of similar temperature measurements, the *γ* values of 6.1 and 6.9 were obtained using data on $E_{X}\left(T\right)$ from [28]. This may be due to the fact that the measurements in [49,50] were carried out in the lasing regime. At the same time, the longer wavelength position of the lasing band as compared with ASE leads to an overestimation of the *γ* value. As an example, in Figure 7, the difference between $E_{X}\left(T\right)$ and the laser band position measured in our case is also plotted. There is also a linear section in this dependence, which can be extrapolated well to zero. However, its slope is larger than in the case of ASE, which gives *γ* = 6.6. Moreover, taking into account the absence of spectrum distortions associated with the appearance of lasing, the ASE spectra should give a more accurate *γ* value.

Lines corresponding to (4) with *n* = 2 (*P*_2_ band) and *n* = ∞ (*P*_∞_ band) and (5) with *γ* = 5.94 are plotted in Figure 5. Together with the experimental behavior of $E_{X-2\mathrm{LO}}\left(T\right)$, they allow one to completely comprehend the optical gain mechanisms in the crystals under study in a wide temperature range up to RT.

At low temperatures, when two bands are observed separately in the ASE spectra of types I and II, the maximum of the high-energy band is within the *P*-band “corridor” between *P*_2_ and *P*_∞_ bands up to *T*~170–180 K. Its deviation towards high energies below *T*~150 K in the type II spectra is due to overlap with the *BX* band and, apparently, with the surface state-related emission. With a further increase in temperature, the maximum of the band goes beyond the *P*-band “corridor”. Thus, we can conclude that the high-energy SE band at temperatures up to ~170–180 K is due to the *X-X* scattering process. In this case, as the temperature rises, the quantum number *n* of the scattered exciton gradually increases towards *n* = ∞ within the *X-X* process. After that, as a result of the partial ionization of excitons, the *X-el* process begins to dominate. Similar observations were made, e.g., in [49], where random lasing was studied in a microcrystalline ZnO film.

The temperature behavior of the low-energy band, which we defined as *X*-2LO, practically does not differ between both types of the ASE spectra below ~200 K. As the temperature increases, the *X*-2LO band merges with the *X-el* band, forming one broad band consisting of these competing emissions. This process is observed up to *T*~250–260 K, after which, in two different types of spectra, i.e., depending on the type of microstructure, resulting emission exhibits the properties of SE of a particular type. Specifically, in the type II spectrum, i.e., in the case of weakly faceted crystals, the *X*-2LO band dominates up to RT. In the case of well-faceted microcrystals (type I spectrum), the resulting band maximum deviates towards lower energies, following the *X-el* process with *γ*~6.

Thus, we believe that the main contribution to RT SE in studied ZnO microrod lasers with WGMs is made by the *X-el* scattering process. The contribution of the *X*-2LO component, which can diminish *γ* value, cannot be ruled out. However, this contribution, especially at pumping intensities above the lasing threshold, should not be decisive. This is evidenced by the temperature dependence of the lasing band energy (Figure 5). Despite the presence of two sets of laser peaks and, consequently, SE of two different types in a certain temperature range simultaneously, the weighted average maximum of the band clearly follows the modeled temperature dependence for the *X-X* process and then only slightly deviates from the *X-el* process. This is confirmed by the calculated difference between $E_{X}\left(T\right)$ and the laser band position (see Figure 7).

## 4. Discussion

In the present study, we show that optical gain with a maximum in the region of 3.11–3.17 eV (391–398 nm) at RT in hexagonal ZnO microrods with WGMs and low lasing thresholds is caused by exciton processes, namely, by the process of *X-el* scattering predominantly, at least at near-threshold excitation intensities. In the region of the sample with weakly faceted crystals, the *X*-2LO mechanism is responsible for the SE at RT; its maximum is in the region near ~3.17 eV (391 nm). Further, the *X*-2LO SE band practically does not shift with increasing pumping, while the *X-el* SE band demonstrates an intense redshift. This shift may be the reason for confusing this type of SE with emission caused by the direct recombination in the inverted EHP, where the redshift is provided mainly by the bandgap renormalization. In the case of the *X-el* process, an increase in the exciton effective temperature can be responsible for the redshift, a relatively large value of which is provided by a comparatively large value of γ.

The existence of excitons, at least at the near-threshold pumping values, is confirmed by a simple estimate of the density of electron-hole (e-h) pairs $n_{p}$ produced by optical interband excitation. For this estimate, one can use the expression $n_{p}=\rho_{exc}\tau_{p}{\left(\hbar \omega_{exc}l\right)}^{-1}$, where $\hbar \omega_{exc}$ is excitation photon energy, $\tau_{p}$ is an e-h pair lifetime, and *l* is the absorption depth. This expression does not take into account the efficiency of creating an e-h pair upon absorption of an excitation photon, as well as the diffusion length of e-h pairs in a crystal, which can reach micron values [51], and therefore gives an upper estimate of $n_{p}$. In our case, using $\tau_{p}$~100 ps, *l*~100 nm [41], such an estimate gives $n_{p}$ no larger than 10^17^ cm^–3^ for near-threshold pumping. This is lower than the Mott transition density for ZnO, which is about 10^18^ cm^−3^, according to the literature data [52,53].

However, in the experiments carried out within the framework of the present study, excitation fluences up to several hundred kW/cm^2^ were used. It cannot be ruled out that, at least at relatively high pumping, the Mott threshold in the studied ZnO microcrystals is exceeded. At the same time, lasing and ASE spectra of the redshifting type with temperature dependence similar to that of the *X-e* process were observed from the sample within the entire photoexcitation range. This means that the gain nature remains the same even after a possible transition to the EHP regime. In this case, according to the works of C.F. Klingshirn et al. [52,54], one can assume that the *X-e* scattering process shifts to the scattering of Coulomb-correlated e-h pairs by free carriers in the EHP. This concept is used to interpret experimental results in some later works, e.g., [17,50]. Moreover, one cannot exclude that the same gain type, instead of direct recombination in the EHP, can also take place in some other cases of WGM lasing in ZnO microcrystals, in particular, microrods, described in the literature, where lasing spectra with similar features are observed in the same spectral range but with higher laser thresholds (e.g., [12,14,15,16]).

It is necessary to note the significant sensitivity of the *X-e* gain mechanism to the crystal perfection. Indeed, despite the similar spectral behavior in the regions of the sample with high-quality microcrystals and with weakly faceted crystals at low temperatures (i.e., the gradual shift in the dominant SE mechanism from *X-X* to *X-el*, followed by the appearance of *X*-2LO SE and its merging with *X-el* emission), at temperatures close to RT, the dominant SE mechanisms are different—in particular, weakly faceted microcrystals do not exhibit *X-el* emission. This emphasizes the enormous influence of the microcrystal shape and surface quality and, as a consequence, the conditions for the optical feedback formation on the excitation thresholds of the *X-el* process and the optical gain for this emission type. In addition, the oriented growth of microrods and, consequently, the absence of contacts with the substrate and other crystals undoubtedly contribute to a decrease in the laser thresholds and an increase in the *Q*-factor as a result of a reduction in optical losses, which can appear in microrods and microwhiskers placed directly on a substrate. At the same time, by not maintaining *X-el* emission near RT, weakly faceted microcrystals made it possible to observe the *X*-2LO band at RT and low temperatures. Minor influence on the *X*-2LO spectral region by other radiative channels and the predominance of the optical gain provided by this emission up to RT allowed us, by using this emission band, to determine $E_{X}\left(T\right)$ in the samples under study. The obtained data on $E_{X}\left(T\right)$ were close to the results of [28]—particularly, they give $E_{g}$ ≅ 3.37 eV at RT—which indicates that the studied microrods exhibit the properties of bulk ZnO crystals. In addition, the obtained dependence $E_{X}\left(T\right)$ allows one to analyze the optical gain mechanisms in crystals. In this context, it is also important to emphasize the role of the joint analysis of data on ASE and lasing. In particular, it made it possible to reveal and identify competing channels of optical gain—*X-el* and *X*-2LO—in the ZnO microstructure studied, which is more difficult to do when analyzing only lasing spectra.

It is worth noting that one should correctly determine $E_{g}$ or $E_{X}$ for a specific sample under study to establish the gain mechanism if the data for bulk/microcrystalline ZnO cannot be used for some reason. In particular, the data on RT spontaneous NBE emission or absorption/reflection should be used carefully. This is due to several factors, including the contribution of *X*-LO emission to the NBE emission band at RT and close temperatures, possible modification of the emission spectrum due to optical modes, and the influence of energy levels of defects, the neglect of which can lead to an underestimation of the $E_{g}$ and $E_{X}$ values. Particularly, if we use $E_{g}$ ~3.36–3.37 eV, the assumption about the presence of the *P* band in the spectral region under consideration can be excluded, even without the results of temperature measurements. In this case, the spectral position between the emission maximum and the free exciton energy exceeds 120 meV, although for the *P* band, this distance should be less than 100 meV according to (4). Moreover, the change of the radiative channel from *X-X* to *X-el* in ZnO in the temperature range of 100–200 K has been reported (see, e.g., [49,52]), including the present work. In particular, the *X-X* process is unlikely to be responsible for the non-shifting or slightly shifting WGM lasing band at RT. The maximum of such a lasing band is usually found within the region of 3.15–3.18 eV at RT, and its thresholds are often rather low, 50–200 kW/cm^2^, under nanosecond interband photoexcitation (see, e.g., [15,18,20,21]). Taking into account our results related to *X*-2LO emission as well as the results of other studies, e.g., [21,52,55], it is quite possible that the *X*-2LO recombination process is the main optical gain mechanism in the case of low-threshold WGM lasing of this type.

## 5. Conclusions

In this work, we studied the mechanisms of optical gain in ZnO microrods with a hexagonal cross-section, which demonstrate WGM lasing in the spectral region of 3.11–3.17 eV (391–399 nm) with a characteristic redshift upon increasing photoexcitation intensity at room temperature. The lasing thresholds observed were down to 10–20 kW/cm^2^ and *Q*-factors were above 3000. It was shown that the main contributor to SE at RT in such microrods, at least at near-threshold excitation intensities, is inelastic exciton-electron scattering. A joint analysis of data on ASE and lasing facilitated the determination of optical gain mechanisms at various temperatures. In particular, at temperatures below RT, the coexistence of two competing processes providing necessary gain for whispering-gallery mode lasing in ZnO microcrystals was revealed: exciton-electron scattering and two-phonon assisted exciton recombination. The latter mechanism was shown to play the main role in the formation of optical gain at RT in weakly faceted ZnO microcrystals, which demonstrate ASE with a maximum in the region of ~3.17 eV without a significant spectral shift. This type of optical gain may be responsible, in particular, for low-threshold WGM lasing in ZnO microstructures that does not exhibit significant redshift, which is reported in the literature.

The results of this work expand our understanding of the nature of optical gain in ZnO, specifically in ZnO microcrystals supporting WGM lasing. In particular, contrary to the widely held opinion about the relationship of SE in WGM ZnO microlasers exhibiting a characteristic redshift at RT upon increasing pumping intensity with direct recombination in an inverted EHP, the present study reports on the possibility of forming an optical gain with a redshifting profile as a result of exciton processes, namely, the process of exciton-electron scattering. Despite the possibility of achieving sufficiently low laser thresholds, emission of this type was observed over a wide excitation range. This indicates the possibility of using this interpretation in the study of similar WGM lasing spectra with a shifting profile instead of direct recombination in the inverted EHP.

## Data Availability

Not applicable.
